# Facile and cost-effective NiO/MgO–SiO_2_ composites for efficient oxygen evolution reaction and asymmetric supercapacitor systems†

**DOI:** 10.1039/d5ra00671f

**Published:** 2025-03-06

**Authors:** Gulzar Ali, Aneela Tahira, Asma Hayat, Mukhtiar Ali Bozdar, Muhammad Ali Bhatti, Elmuez Dawi, Ayman Nafady, Matteo Tonezzer, Ghulam Mustafa Thebo, Muhammad Kashif Samoon, Zafar Hussain Ibupoto

**Affiliations:** a Institute of Chemistry, University of Sindh Jamshoro 76080 Pakistan gulzaralichemist@gmail.com asmabaloch141617@gmail.com zaffar.ibhupoto@usindh.edu.pk; b Institute of Chemistry, Shah Abdul Latif University Khairpur Mirs Sindh Pakistan aneela.tahira@salu.edu.pk; c Centre for Environmental Sciences, University of Sindh Jamshoro Sindh 76080 Pakistan mali.bhatti@usindh.edu.pk; d College of Humanities and Sciences, Department of Mathematics and Sciences, Ajman University P. O. Box 346 Ajman United Arab Emirates e.dawi@ajman.ac.ae; e Chemistry Department, College of Science, King Saud University Riyadh 11451 Saudi Arabia anafady@ksu.edu.sa ralshammari@ksu.edu.sa; f Department of Chemical and Geological Sciences, University of Cagliari Monserrato Italy matteo.tonezzer@cnr.it; g Centre for Pure and Applied Geology, University of Sindh Jamshoro Jamshoro Sindh 76080 Pakistan kashif.samoon@usindh.edu.pk; h Department of Energy and Environment, Sindh Agriculture University Tando Jam Sindh Pakistan

## Abstract

Biomass waste from grapefruit peel extract was used for the preparation of MgO–SiO_2_ composites *in situ* in order to develop effective electrocatalytic composites based on NiO/MgO–SiO_2_. The MgO–SiO_2_ composites were subsequently deposited with NiO using a modified hydrothermal method. The synthesized materials were analyzed to investigate their morphology, crystal structure, chemical composition, functional group, and optical band gap. The structural analysis allowed us to determine the orientation of the nanoparticles, the cubic phase of NiO and MgO, the significant loss of optical band gap, and the enriched functional groups on the surface of NiO/MgO–SiO_2_ composites. The electrochemical properties were investigated in the presence of an alkaline solution of KOH. To study the oxygen evolution reaction (OER) in 1 M KOH aqueous solution, different NiO/MgO–SiO_2_ composites were investigated. It was found that the NiO/MgO–SiO_2_ composite that contained the highest amount of MgO–SiO_2_ (sample 3) had a lower overpotential than the NiO/MgO–SiO_2_ composite with the lowest amount of MgO–SiO_2_. Sample 3 exhibited an overpotential of 230 mV at 10 mA cm^−2^ over a period of 40 hours with excellent stability. The superior electrochemical activity of the NiO/MgO–SiO_2_ composite (sample 3) was demonstrated in an energy storage device using 3 M KOH aqueous solution, and asymmetric supercapacitor devices were fabricated in 3 M KOH solution. According to the ASC's specifications, a specific capacitance of 344.12 F g^−1^ and an energy density of 7.31 W h kg^−1^ were found for the device at a fixed current density of 1.5 A g^−1^. After over 40 000 galvanic charge–discharge repeatable cycles at 1.5 A g^−1^, sample 3 of the NiO/MgO–SiO_2_ composite exhibited excellent cycling stability with 88.9% percent capacitance retention. During the performance evaluation of the NiO/MgO–SiO_2_ composites, grapefruit peel extract was confirmed as a potential biomass waste for the fabrication of high-performance energy conversion and storage devices.

## Abstract 2

Biomass waste from grapefruit peel extract was used for the preparation of MgO–SiO_2_ composites *in situ* in order to develop effective electrocatalytic composites based on NiO/MgO–SiO_2_.

## Graphical abstract

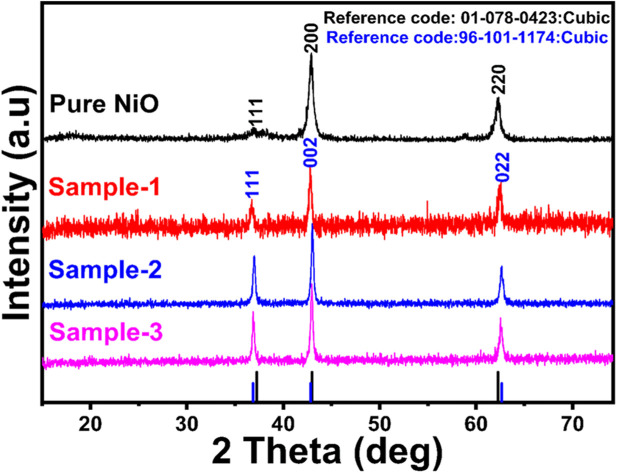

## 1. Introduction

The burning of fossil fuels has resulted in severe environmental consequences and led to a global energy crisis due to resource depletion. Researchers have developed clean and eco-friendly renewable energy resources as potential alternatives to fossil fuels.^1,2^ One such clean energy source, hydrogen (H_2_) has received significant attention due to its high energy density, cleanliness, and environmental friendliness, which makes it an attractive replacement for fossil fuels.^3^ Hydrogen can be produced by electrochemical water splitting, which has been found to be an innovative and attractive technology.^4^ Two half-cell reactions occur during electrochemical water splitting, namely the hydrogen evolution reaction (2H_2_O + 2e^−^ → H_2_ + 2OH^−^) at the cathode and oxygen evolution reaction (4OH^−^ → O_2_ + 2H_2_O + 4e^−^) at the anode.^5^ As clean energy sources, energy storage devices must be able to harvest and store energy for use at any time.^6–8^ This has led to a growing number of studies focusing on the development of efficient energy conversion and storage systems, such as fuel cells, supercapacitors, and batteries.^9–11^ Due to its high power density, long cycling stability, and ability to work at high rates, the supercapacitor has been identified as an energy storage system to be investigated. In addition, supercapacitors have a low maintenance cost and a rapid rate of charge mobility. Evidence suggests that supercapacitors serve as an intermediary between capacitors with high power densities and batteries with high energy densities.^12–14^ Compared to batteries, supercapacitors have a relatively low specific energy density. Since these types of energy storage devices can be used in a wide range of applications, such as memory backup devices, power grids, hybrid electric vehicles, and other related applications, much effort has been devoted to improving the performance of these types of energy storage devices.^15^ A supercapacitor can be classified into two categories based on its working principle: electric double-layer capacitors (EDLCs) and pseudo capacitors.^16^ Pseudo capacitors store charge by faradaic processes, while EDLCs store charge by electrostatic processes. Pseudo capacitors have been made from a variety of electrode materials, including transition metal oxides (RuO_2_, NiO, MnO_2_, and ZnO),^17^ metal sulfides,^18^ and conducting polymers such as polyaniline (PANI).^19,20^ These electrode materials display rapid, reversible faradaic reactions and offer ten to one hundred times greater specific capacitance than EDLC.^21^ Ruthenium oxide (RuO_2_) is one of the most popular transition metal oxide electrode materials due to its excellent redox chemistry and high electrochemical activity.^22,23^ Considering these two half-cell reactions, the OER process is highly energy-demanding and kinetically slow as a result of the four electron transfer steps.^5^ Furthermore, researchers have been motivated to find and develop low-cost and earth-abundant electrode materials to address the high cost, scarcity, and significant toxicity of RuO_2_. NiO has been found to exhibit excellent redox reversibility as well as a high specific capacitance (2584 F g^−1^)^24^ and low manufacturing costs.^25^ NiO is subject to ion exchange mechanisms,^26^ so its specific capacitance decreases rapidly with increasing scan frequency, thereby reducing its performance. There are large volumetric changes in the NiO molecule during charge–discharge cycling, which results in passivation and rapid charge decay.^26^ NiO exhibits a low level of electrochemical activity in addition to its poor electrical conductivity. A number of attempts have been made to improve the electrode performance of NiO during electrochemical water-splitting supercapacitors^27^ in order to minimize these drawbacks. Magnesium oxide (MgO) has been used as a refractory material and as a catalyst for heterogeneous catalysis since it is inexpensive, earth-abundant, and eco-friendly.^28,29^ The wide band gap of magnesium oxide makes it less suitable for use in electrochemical energy storage systems.^30^ Magnesium oxide has been shown to contribute significantly to the overall life of energy storage electrode materials, both as a stabilizing agent and matrix element.^30^ The MgO used in this case acts as a stabilizing agent for the NiO based electrode material, thus minimizing the issues related to cycling stability and volume expansion during the charge–discharge cycle, resulting in improved electrode performance. Furthermore, the use of silica (SiO_2_) gel can significantly enhance the performance of energy storage systems, as well as influence the microstructure and particle size of the composite electrode material. Therefore, a number of studies have demonstrated that SiO_2_-based composite electrode materials are suitable for the development of supercapacitors.^31^

By increasing the redox activity of SiO_2_, the specific capacitance of composite electrode materials was increased, as well as the surface area and cycling stability. The combination of metal oxides with SiO_2_ has been shown to provide unique shape architectures, crystal structures, and redox-active sites for improving the electrochemical performance of composite electrode materials.^32–35^ The shortcomings of NiO as an electrode material include poor electrical conductivity, limited catalytic sites, and poor cycling stability. We propose using MgO and SiO_2_ as stabilizing agents, excellent matrix elements, agents for increasing surface area, and agents for enhancing redox sites, to produce the first NiO/MgO–SiO_2_ composite. A composite of MgO and SiO_2_ was prepared *in situ* using grapefruit peel extract *via* a modified hydrothermal method. The biomass waste of industrial grapefruit juice production has been used for food and nonfood-related applications. It can be employed as a natural resource for medicines, cosmetics, packaging materials, and biofuels. Grapefruit peel contains various phytochemicals such as terpenoids, flavonoids, and quinolines. These phytochemicals exhibit structure-directing, reducing and stabilizing agent properties, thus rendering grapefruit an effective green resource for modifying the surface and functional properties of materials. The phytochemicals from grapefruit peel extract were fully exploited in order to modify the surface, align the structure, and enrich the redox sites of composite materials. We developed NiO-based composite electrodes for OER and supercapacitors using MgO–SiO_2_ composites of differing amounts deposited during hydrothermal processes. There has been no previous research on the role played by varying amounts of MgO–SiO_2_ grown *in situ* during the fabrication of NiO composite configurations based on MgO–SiO_2_.

## 2. Materials and methods

### 2.1. Chemical reagents

Sigma Aldrich, Sindh Karachi Pakistan, provided aqueous ammonia solution (33%), magnesium nitrate hexahydrate, nickel chloride hexahydrate, ethanol (99.5%), acetone (99.9%), silica gel, ethanol (99.5%), urea (99%), Nafion 5%, and potassium hydroxide (99%). All of these items were used without further treatment. Aqueous solutions were prepared using deionized water. Grapefruit peels were removed from fresh fruit purchased from a local market.

### 2.2. Preparation of NiO/MgO–SiO_2_ composites

A deionized water solution was applied to the grapefruit, followed by successive drying at room temperature. The peel of the grapefruit was removed and crushed into fine pieces, and a mass of 200 grams was dispersed in 500 mL of deionized water. During the next 24 hours, the fine peel pieces were continuously stirred. After the peel extract had been obtained, the extract was filtered using standard laboratory filter paper. Next, 200 mL of grapefruit peel extract was mixed with 0.5 M magnesium nitrate hexahydrate and silica gel. Using concentrated aqueous ammonia solution, the pH of the solution was maintained at around 10 during the growth process, which took place at 80 °C for a period of 2–3 hours. The product was then collected onto a filter paper and dried at room temperature for two hours. After the powder had been phased in a crucible for four hours, it was thermally burnt at 600 °C. The resulting composite was white in color and contained MgO–SiO_2_. A NiO/MgO–SiO_2_ composite was developed using 0.1 g (sample 2) and 0.3 g (sample 3) of MgO–SiO_2_ composite in 100 mL of deionized water containing 0.1 M nickel chloride hexahydrate and urea. A sheet of aluminum was used to cover the growth solutions. The hydrothermal process took place at 95 °C for five hours. After receiving the product, it was washed several times with deionized water and thermally treated at 500 °C for five hours. In the end, it was found that NiO composites with MgO and SiO_2_ were successfully formed. In the same process, pure NiO was obtained without the use of MgO–SiO_2_ composites. An examination of the morphology of the as-synthesized materials was conducted using scanning electron microscopy at a voltage of 10 kV. A PAnalytic X'Pert PRO X-ray diffractometer was used to analyze the crystal systems within the prepared materials under the operating conditions of 10 to 80 two theta, 0.02 step, and 0.3 seconds. A UV-visible spectrophotometer was used to collect absorption spectra in the wavelength range of 200–700 nm. An analysis of the surface functional groups carried by the prepared materials was conducted using Fourier transform infrared (FTIR) spectroscopy in the frequency range of 4000–400 cm^−1^ by employing KBr pellets.

### 2.3. Electrochemical characterization

The electrochemical tests were performed using a Versa potentiostat. For analyzing the OER activity of the as-synthesized materials, different electrodes, including a glassy carbon electrode (GCE) as the working electrode, platinum wire as the counter electrode, and silver–silver chloride (Ag/AgCl, filled with 3 M KCl solution) as the reference electrode, were used. GCDE was polished using alumna paste and silicon paper, followed by washing with deionized water. We prepared the catalyst slurry by mixing 5 mg of each material in 2–3 mL of deionized water and 100 microliters of 5% Nafion, followed by ultrasonication in an ultrasonic bath for 15–20 minutes. A 5 microliter catalyst slurry was applied to GCDE with a loading mass of 0.2 mg and dried at room temperature. A 1 M KOH aqueous solution was used to evaluate OER performance. The materials were deposited onto nickel foam as porous electrodes for the analysis of supercapacitors. Nickel foam has a high adsorption capacity for hydroxide ions and good current collection properties. To ensure good adhesion, the material was deposited on the nickel foam using the dip coating method followed by mild annealing at 70–75 °C for 20 minutes. For the purpose of facilitating the high adsorption rate of hydroxide ions onto the electrode materials, a 3 M KOH electrolyte was used. A variety of electrochemical methods were used, including cyclic voltammetry (CV), linear sweep voltammetry (LSV), chronoamperometry, and galvanic charge discharge (GCD). EIS study was conducted between the frequencies of 100 kHz and 0.1 Hz, with amplitudes of 10 mV and biasing potentials of onset potentials of OERs.

## 3. Results and discussion

### 3.1. Structural analysis of the NiO/MgO–SiO_2_ composites

**Fig. 1:**
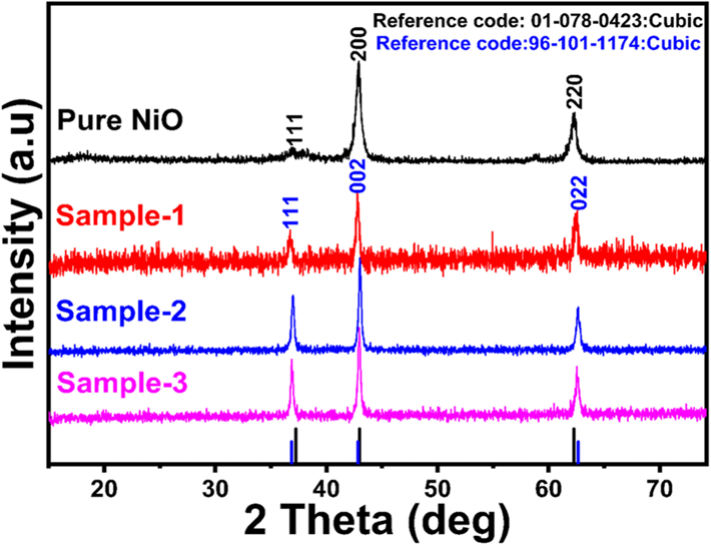
Diffraction patterns measured by XRD for pure NiO and NiO/MgO–SiO_2_ composites (sample 1 and sample 2).

Powder XRD patterns of pure NiO, MgO–SiO_2_ composite and different NiO/MgO–SiO_2_ composites were collected, as shown in Fig. 1. The reflections at two theta values of 37.85, 41.98, and 63.44 correspond to crystal planes such as (111), (200), and (220). As NiO and MgO have similar reflections at two-theta, we were unable to observe much difference between the XRD results of the two materials. In the NiO/MgO–SiO_2_ composites, NiO and MgO were in the cubic phase, with no other phases or impurities present. A high amorphous contribution of silica prevented the detection of patterns. Consequently, all NiO/MgO–SiO_2_ composites were characterized by the presence of cubic phases.

**Fig. 2:**
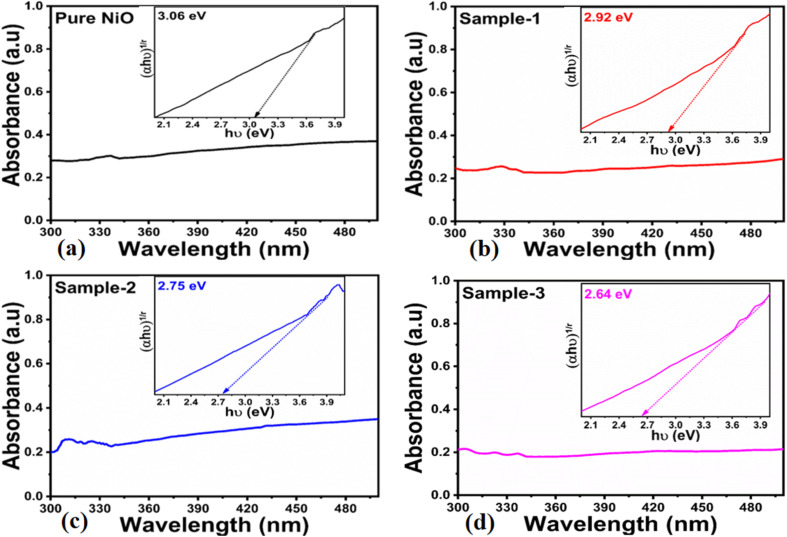
UV-visible absorption spectra of (a) pure NiO and (b–d) NiO/MgO–SiO_2_ composites (sample 1 and sample 2).

The UV-visible absorption spectra of pure NiO and various NiO/MgO–SiO_2_ composites are shown in Fig. 2. The wavelength range of 200–700 nm was employed and the typical peak was noticed at 350 nm due to the inter-band π–π* electronic transition. The composites displayed two broad absorption peaks at 290 nm and 350 nm. According to the results, the composite of NiO/MgO–SiO_2_ prepared with the highest amount of MgO–SiO_2_ resulted in a shift to longer wavelengths, indicating the possibility of variation in the optical band gap.

A Tauc plot^36^ was used to estimate the optical band gap of each material, as shown in eqn (1).

1 (*αhν*)*n* = *A*(*hν* − *E*_g_)

Here, *α* represents the absorption coefficient, *A* describes the proportionality constant, *hν* represents the photon energy, *E*_g_ is the energy band gap and *n* indicates the type of transition occurring during photon interaction with the specific material. The optical band gap decreased with successive additions of MgO–SiO_2_ during the growth process, as shown in the inset in Fig. 2a–d. It was estimated that the optical band gaps of pure NiO and different NiO/MgO–SiO_2_ composites were 3.06, 2.92, 2.75, and 2.64 eV, respectively. Generally, it is accepted that changes in the synthetic method, particle size, shape orientation, and crystal defects may alter the optical band gap, but the exact physical mechanism underlying the reduction is not yet clear.^37^

**Fig. 3:**
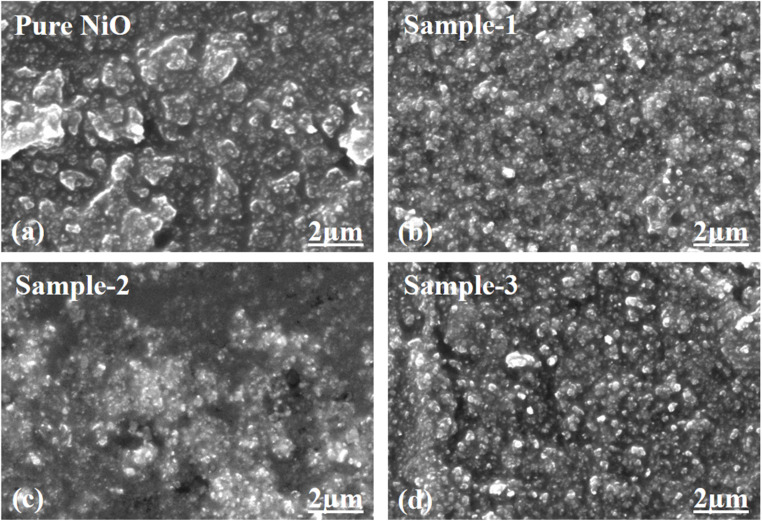
Distinctive SEM images of (a) pure NiO, (b) MgO–SiO_2_ composites (sample 1) and (c and d) NiO/MgO–SiO_2_ composites (sample 2 and sample 3).

**Fig. 4:**
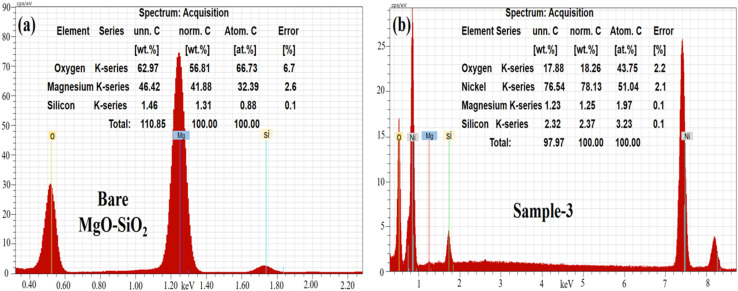
EDX spectra of the (a) bare MgO–SiO_2_ composite and (b) NiO/MgO–SiO_2_ composites (sample 3).

**Fig. 5:**
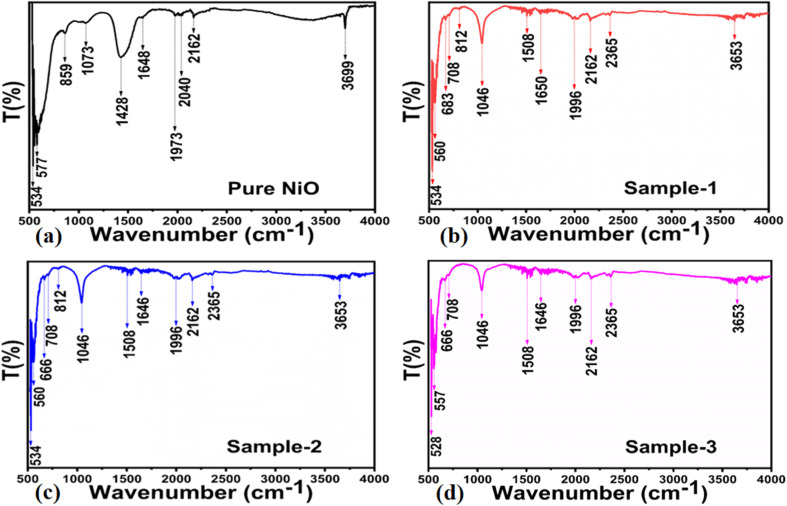
FTR spectra of (a) pure NiO, (b) MgO–SiO_2_ composites (sample 1), and (c and d) NiO/MgO–SiO_2_ composites (sample 2 and sample 3).

The as-synthesized pure NiO and NiO/MgO–SiO_2_ composites were examined using scanning electron microscopy (SEM), and the distinctive images of each sample are presented in Fig. 3. The pure NiO exhibited a petal-like leaf morphology with heterogeneous sizes, as shown in Fig. 3a. As well as the shape structure of NiO/MgO–SiO_2_ composites, the synthesis of these materials using 0.1 g, 0.2 g, and 0.3 g of MgO–SiO_2_ was studied, and the corresponding SEM images are depicted in Fig. 3b–d. The addition of MgO–SiO_2_ altered the shape orientation of NiO, and the resulting composites had a typical nanoparticle structure with uniform distribution and sizes between 100 and 200 nm. As shown in Fig. 3b–d, there was a significant effect of MgO–SiO_2_ on the morphology. The morphology of the NiO/MgO–SiO_2_ composites can be explained by the substrate role of MgO–SiO_2_, hence, the MgO–SiO_2_ composite dominated the nanoparticles' shape orientation. As the nanoparticle shape of the MgO–SiO_2_ composite could be covered with a thin layer of NiO, we did not observe any significant evolution in the NiO morphology in the MgO–SiO_2_ composite. Furthermore, energy dispersive spectroscopy (EDS) was used to locate and determine the nature of the elements in MgO–SiO_2_ and NiO/MgO–SiO_2_ (sample 3), as shown in Fig. 4a and b. It was observed that the elemental composition exhibited by MgO–SiO_2_ was supported by Mg, O and Si as the main elements as shown in Fig. 4a. However, in the NiO/MgO–SiO_2_ composite (sample 3), Ni, O, Mg, Si were the main constituents, as shown in Fig. 4b. In Fig. 5, the surface functional groups of NiO and NiO/MgO–SiO_2_ composites were analyzed by FTIR in the IR region of 200–4000 cm^−1^. There are several IR bands exhibited by pure NiO, such as 440 cm^−1^ and 660 cm^−1^, that are characteristic of the Ni–O and Mg–O stretching vibration bands.^38,39^ Based on the broadness of IR bands, NiO appears to be crystallized. Absorption bands in the vicinity of 2300 cm^−1^ could be attributed to the asymmetric vibrations of carbon dioxide that emerge from the environment.^40^ Since the IR measurements were conducted in air, the typical IR bands at 3400 cm^−1^, 1400 cm^−1^, and 1600 cm^−1^ might be related to the stretching and bending vibrations of the –OH groups absorbed by the surface of NiO from the environment.^40^ The vibration frequency at around 800 cm^−1^ could be attributed to the silica nanoparticles. Bands at 900 cm^−1^ and 1100 cm^−1^ are associated with tensile vibrations in Si–O–Si.^41^ The IR study revealed the presence of SiO_2_, MgO and NiO as the main constituents in the NiO/MgO–SiO_2_ composites.

### 3.2. Enhanced oxygen evolution reaction performance of the NiO/MgO–SiO_2_ composites

**Fig. 6:**
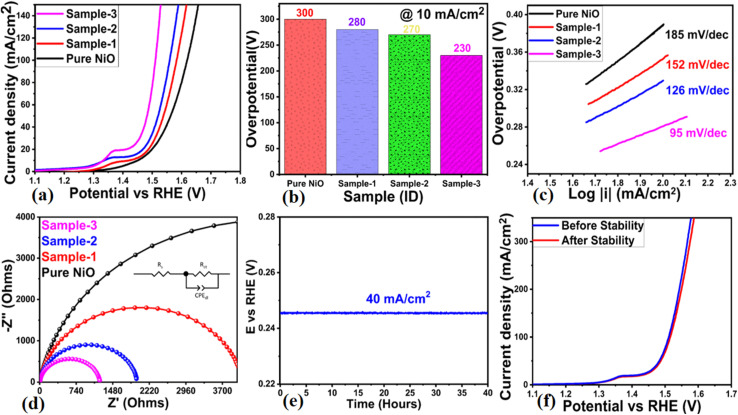
(a) LSV curves measured at 2 mV s^−1^ for bare MgO–SiO_2_ (sample 1), NiO/MgO–SiO_2_ composites (sample 2 and sample 3) in aqueous 1 M KOH electrolyte, (b) bar graph representation for overpotentials, (c) corresponding Tafel analysis on measured LSV curves, (d) Nyquist plots of pure NiO, bare Co_3_O_4_/MgO–SiO_2_ composite (sample 1), and NiO/MgO–SiO_2_ composites (sample 2 and sample 3) using EIS measurements in a frequency range of 100 kHz to 0.1 Hz, at 10 mV amplitude and OER onset potential, (e) chronoamperometry measurements for 40 hours at a fixed current density of 40 mA cm^−2^, and (f) stability expression *via* LSV curves obtained at 2 mV s^−1^ after and before chronoamperometry tests.

A variety of materials, including pure NiO, bare MgO–SiO_2_ composites, and NiO/MgO–SiO_2_ composites containing 0.1 g and 0.3 g of MgO–SiO_2_ composites (sample 2, sample 3), were investigated for their OER activity in 1 M KOH electrolytic solution. To stabilize the electrode, CV was swept at a slow scan rate of 5 mV s^−1^ prior to OER characterization *via* LSV. Afterwards, LSV was swept at 2 mV s^−1^ in 1 M KOH for the examination of OER activity of pure NiO, bare MgO–SiO_2_ composites and their various NiO/MgO–SiO_2_ composites (sample 2, sample 3). According to Fig. 6a, sample 2 had a relatively higher OER activity at a low onset potential than pure NiO, bare MgO–SiO_2_ composite, and sample 2. Further, a figure of merit analysis was performed on all of the tested samples based on their overpotential, and the corresponding values are shown in Fig. 6b. Clearly, the overpotential of sample 3 was smaller than that of pure NiO, bare MgO–SiO_2_ composite, and sample 1, indicating the composite's functional role in accelerating NiO electrochemical activity through the development of multiple surface active sites, charge transport and structural mechanical stability, as is evident from Fig. 6a LSV curves. Based on the bar graph shown in Fig. 6b, it is clear that sample 3 has an overpotential of 230 mV, lower than pure NiO, bare MgO–SiO_2_ composite, and sample 2, which have overpotentials of 300 mV, 280 mV, and 270 mV respectively. The addition of the MgO–SiO_2_ composite to NiO/MgO–SiO_2_ composites increased OER activity by reducing overpotential.

Tafel analysis was conducted using straight-line plots of overpotential against the logarithm of current density. In Fig. 6c, the Tafel slopes of pure NiO, bare MgO–SiO_2_ composite and samples 2 and 3 of NiO/MgO–SiO_2_ composites are shown. Tafel slopes of the presented electrocatalysts were compared with those of Ni-based materials.^42–45^ The observed values were comparable to or lower than those of the previously employed electrocatalysts, indicating that the MgO–SiO_2_ composite may have a positive effect on NiO's Tafel slope value. Due to the possible synergetic effect between NiO and MgO–SiO_2_ composite, and the facilitation of charge transport *via* the reduced band gap of sample 3, favorable OER kinetics have been demonstrated by NiO/MgO–SiO_2_ composites. The MgO–SiO_2_ composite offers possible variability in the local catalytic environment of NiO nanostructures for accelerating the electrochemical OER activity. Also, it provides the nano surface for the possible optimum exposure of catalytic sites of NiO and tunes the charge transport properties at the interface of electrode and electrolyte for rapid electrochemical reactions. In order to support the LSV, overpotential, and Tafel slope results of the electrocatalysts presented, an EIS analysis was carried out. As illustrated in Fig. 6d, the fitted Nyquist plots were obtained through simulation using the Z-View software. There was a characteristic occurrence of a single semicircle at the fixed biasing onset potential for each electrocatalyst, indicating the kinetics of charge transfer between the electrode and electrolyte.^46^ EIS data was fitted using the equivalent circuit with well-resolved circuit elements such as solution resistance (*R*_s_), charge transfer resistance (*R*_ct_), and double-layer capacitance (*C*_dl_). In the case of pure NiO, bare MgO–SiO_2_ composites, and samples 2 and 3 of NiO/MgO–SiO_2_ composites, the charge transfer resistance values were 9161, 4093, 1969 and 1214 Ω, respectively. Based on the effect on particle size, shape orientation, synergetic effect and excellent material compatibility with the electrode surface, it was observed that the charge transfer resistance was linearly decreased as the MgO–SiO_2_ composite was added into NiO/MgO–SiO_2_ composite synthesis. As a result, the electrode and electrolyte were able to transfer charge quickly. An important parameter to evaluate the suitability of a real-time electrolyzer device is the stability of the electrocatalyst. Consequently, the stability of sample 3 based on NiO/MgO–SiO_2_ composite was investigated using a fixed current density of 40 mA cm^−2^ for 40 hours as shown in Fig. 6e. Sample 3 showed a minute loss of overpotential at fixed current density, suggesting its good compatibility with the electrode. It is likely that sample 3 exhibited improved stability due to the mechanical stability that was conferred by the bare MgO–SiO_2_ composite into NiO/MgO–SiO_2_ composite *via* a unique structure architecture and the easy bubbling of oxygen gas out of the composite. The durability of sample 3 of the NiO/MgO–SiO_2_ composite was evaluated by comparing the LSV curves measured before and after the stability test for 40 hours, as shown in Fig. 6f. Sample 2's LSV curves overlapped without any significant differences in onset potential or overpotential, demonstrating its excellent durability and capability for long-term use. An electrocatalyst's performance is directly related to its surface area that favors OER activity due to the exposure of active sites to the electrolyte, resulting in an enhanced oxidation process.^47^ As shown in the ESI Fig. S1,† the electrochemical active surface area (ECSA) was calculated by measuring the double layer capacitance (*C*_dl_) using non-faradic CV curves for pure NiO, the bare MgO–SiO_2_ composite, and samples 2 and 3 of the NiO/MgO–SiO_2_ composite at varying scanning rates. In the CV curves, the linear shape was verified against different scan rates with significant slope values corresponding to *C*_dl_ of each electrocatalyst. The current density was picked for the anodic and cathodic sides at a fixed potential of 0.02 V against Ag/AgCl and in this potential range, the CV behavior was found to be almost non-faradic in nature. In order to obtain the ECSA values of these materials, the current density was divided by the electrode area.^47^ The ECAS analysis indicated the corresponding values of pure NiO, bare MgO–SiO_2_ composite, sample 1 and sample 2 as 0.118, 0.009, 0.120 and 0.128 mF cm^−2^ respectively. Electrocatalytic performance could be improved due to the enriched surface area in sample 3. Moreover, the performance of the NiO/MgO–SiO_2_ composite-based sample 3 towards OER was compared with recent reports and observations are given in ESI Table S1.† It was observed that the presented NiO/MgO–SiO_2_ composite is low-cost, facile, exhibits a low overpotential and is ecofriendly, hence it could be used as an alternative electrocatalyst for water splitting in alkaline conditions. In alkaline electrolyte conditions, hydroxide ions are adsorbed on the surface of the electrode for the promotion of OER half-cell water splitting. Generally, the adsorption of hydroxide ions on the surface of metal oxide involves several steps with successive release of oxygen gas. For instance, OER proceeds in alkaline electrolytes *via* the following sequential steps eqn (2)–(5):

2 OH^−^ → (OH)_ads_ + e^−^

3 (OH)_ads_ + OH^−^ → (O^−^)_ads_ + H_2_O

4 (O^−^)_ads_ → (O)_ads_ + e^−^

5 2(O)_ads_ → O_2_↑

### 3.3. Supercapacitor performance of the NiO/MgO–SiO_2_ composites

**Fig. 7:**
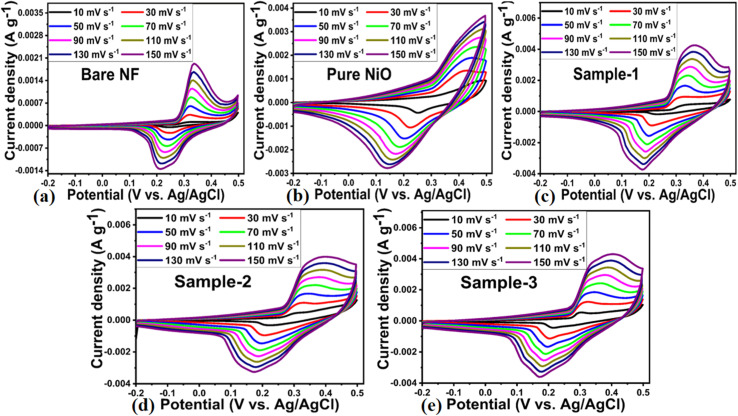
CV curves of (a) bare nickel foam, (b) pure NiO, (c) MgO–SiO_2_ composites (sample 1), and (d and e) NiO/MgO–SiO_2_ composites (sample 2 and sample 3) using different scan rates in electrolytic solution of 3 M KOH for the illustration of capacitance performance of each electrode material.

From the structural and electrochemical OER investigations, it was found that the inclusion of the MgO–SiO_2_ composite during NiO/MgO–SiO_2_ composite preparation increased structure variation, particle size, and surface functionality, thereby enhancing electrochemical performance during OER activity. NiO/MgO–SiO_2_ composite samples 2 and 3 were deposited onto nickel foam due to its large surface area and excellent current collection properties and used during CV measurements in 3 M KOH aqueous solution. During CV experiments, the potential window was −0.1 to 0.6 V and different scan rates were employed. As shown in Fig. 7a–e, the corresponding curves for nickel foam, pure NiO, bare MgO–SiO_2_ composite, sample 2 and sample 3 were recorded at various scan rates. As a result of the use of electrode materials, two distinctive redox peaks were observed, indicating the features of pseudocapacitance energy storage. A typical reversible energy storage system between Ni^2+^ and Ni^3+^ is represented in the following chemical equation.^48^

6 NiO + OH^−^ ⇌ NiOOH + e^−^

**Fig. 8:**
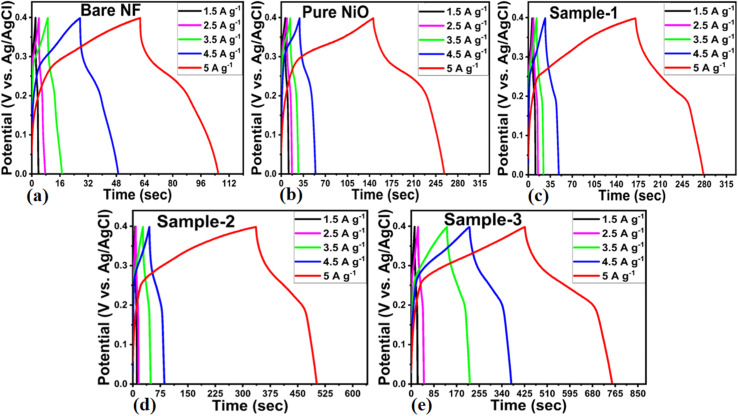
Galvanic charge–discharge cycles of (a) bare nickel foam, (b) pure NiO, (c) MgO–SiO_2_ composites (sample 1), (d and e) NiO/MgO–SiO_2_ composites (sample 2 and sample 3) using current densities from 1.5 to 5 A g^−1^ in an electrolytic solution of 3 M KOH for the illustration of capacitance performance of each electrode material.

**Fig. 9:**
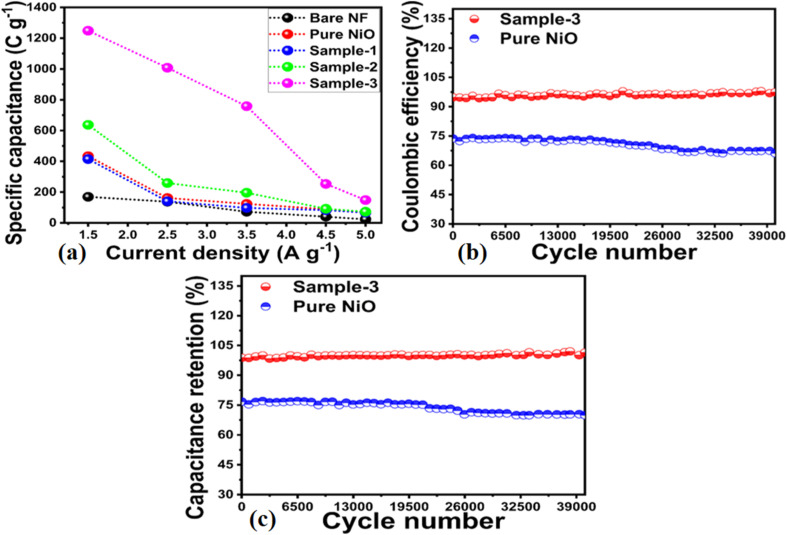
(a) Calculated specific capacitance MgO–SiO_2_ (sample 1), NiO/MgO–SiO_2_ composites (sample 2 and sample 3) from GCD cycles at different current densities of 1.5 A g^−1^ to 5 A g^−1^, (b) corresponding coulombic efficiency analysis for repeatable GCD cycles of 40 000 at 1.5 A g^−1^ for pure NiO and NiO/MgO–SiO_2_ composites (sample 3), and (c) % capacitance analysis for repeatable GCD cycles of 40 000 at 1.5 A g^−1^ for pure NiO and NiO/MgO–SiO_2_ composites (sample 3).

The CV curve analysis revealed that sample 1 retained the well-defined redox peaks without significant alteration at high scan rates, suggesting good electrochemical activity and cycling rate. NiO/MgO–SiO_2_ composite sample 3 exhibited CV curves that were relatively larger than those of other electrode materials, thus confirming the superior electrochemical capacitance at the lowest scan rate of 5 mV s^−1^. CV curves demonstrated high correlation with electrochemical results obtained during OER studies, confirming the enhanced electrochemical activity of sample 3. As a result of the large surface area, unique mechanical structure, rapid charge transport, and synergetic effect, sample 3 demonstrated enhanced pseudocapacitive energy storage performance over bare nickel foam, pure NiO, bare MgO–SiO_2_, and sample 1 of NiO/MgO–SiO_2_. MgO–SiO_2_ composites with high content provided a large nanoscale surface area for the deposition of NiO layers, thus increasing the surface area of the NiO/MgO–SiO_2_ composite for frequent interaction with hydroxide ions, resulting in high adsorption of hydroxide ions and therefore fast charge storage. A subsequent increase in the current was seen in the CV curves with increasing scan rates as a result of the development of thick diffusion layers at slower scan rates, which restricted the electrolyte flow penetrating the electrode,^49^ hence the decrease in current as depicted in Fig. 7a–e. As the scan rate is increased, irreversibility and quasi-reversibility increase because of potential polarization and electrolytic resistance, which further shifts oxidation and reduction peaks to higher and lower potentials, respectively.^50^ CV curves of sample 2 and sample 3 of NiO/MgO–SiO_2_ composites indicated more battery-like characteristics. CV curves for sample 3 show a higher current response than that of sample 2, indicating that both the MgO–SiO_2_ composite and NiO exhibited synergetic effects. Furthermore, the higher content of the bare MgO–SiO_2_ composite during the preparation of sample 3 remained responsible for the generation of current due to differences in the synthetic conditions. Galvanic charge–discharge (GCD) analysis was carried out to describe the electrode materials' electrochemical performance using different current densities in 3 M KOH electrolytic solution as shown in Fig. 8a–e. A variety of electrode materials were used, including pure NiO, bare nickel foam, bare MgO–SiO_2_ composite, and samples 2 and 3 of NiO/MgO–SiO_2_ composite. They were tested in GCD measurements at current densities of 1.5, 2.5, 3.5, 4.5, and 5.5 A g^−1^, as shown in Fig. 8a–e. GCD curves indicated the pseudo capacitance features of electrode materials to be used; however, sample 3 exhibited superior specific capacitance and was highly correlated with the CV curves. Furthermore, the electrode material based on sample 3 has the largest charging–discharging cycles as compared to pure NiO, bare nickel foam, bare MgO–SiO_2_ composite and sample 2 of NiO/MgO–SiO_2_ composite. Using a previously reported method^48^, the specific capacitance (*C*_s_) of these electrode materials was estimated at different current densities. As shown in Fig. 9a, the resultant *C*_s_ values for pure NiO, bare nickel foam, bare MgO–SiO_2_ composite, and samples 2 and 3 of NiO/MgO–SiO_2_ composite were determined. Among the electrode materials, sample 2 had the highest *C*_s_ at 1.5 A g^−1^, with the lowest current density. The inclusion of MgO–SiO_2_ composite into NiO for developing NiO/MgO–SiO_2_ composite plays a significant role in enhancing the specific capacitance due to the larger surface area, higher adsorption rate of hydroxide ions, and rapid charge transfer. In sample 3, the solution resistance decreased as a result of enhanced ion transport at the electrode/electrolyte interface. Due to its high electrochemical performance, sample 3 has been used for stability testing using repeatable 40 000 GCD cycles at 1.25 A g^−1^ and compared with the pure NiO for demonstrating cycling stability in terms of capacitance retention % and coulombic efficiency, as shown in Fig. 9b and c. It could be seen the pure NiO suffered from poor % capacitance retention and coulombic efficiency compared to NiO/MgO–SiO_2_ (sample 3). During 40 000 GCD cycles, sample 3 of NiO/MgO–SiO_2_ composite maintained 101.5% of its initial capacitance and nearly 97.6% coulombic efficiency. In the repeatable GCD cycles, sample 3 was subjected to mechanical stress and strain as well as electrolytic ions extraction at all times during stability measurements, which contributed to slight degradation.^51^ Briefly, the obtained results of one electrode material including bare nickel foam, pure NiO, MgO–SiO_2_ composite (sample 1), NiO/MgO–SiO_2_ composite (samples 2 and 3) are given in ESI Table S2.† Also, the performance of the NiO/MgO–SiO_2_ composite (sample 3) was compared with the reported electrode materials in terms of specific capacitance as given in ESI Table S3.† It was clearly observed that the presented electrode material has a high potential to store the charge owing to its facile and ecofriendly nature.

### 3.4. Asymmetric supercapacitor (ASC) device application of the NiO/MgO–SiO_2_ composite

**Fig. 10:**
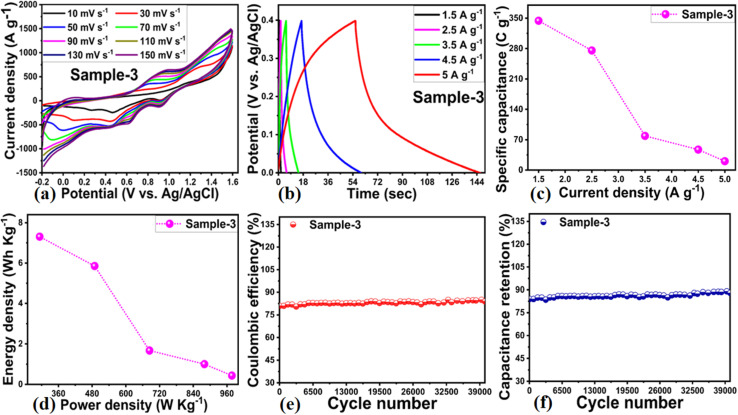
(a) ASC performance of NiO/MgO–SiO_2_ composites (sample 3) as the anode material and cathode of AC through CV cycles at different scan rates in 3 M KOH electrolytic solution, (b) GCD curves of NiO/MgO–SiO_2_ composites (sample 3) ASC, (c) calculated specific capacitance NiO/MgO–SiO_2_ composites (sample 3) through GCD cycles measured at various current densities, (d) corresponding energy and power density of NiO/MgO–SiO_2_ composite (sample 3) as the anode material during ASC device, (e) ASC cycling stability performance of the NiO/MgO–SiO_2_ composite (sample 3) during 40 000 GCD repeatable cycles at fixed current density of 1.5 A g^−1^ through coulombic efficiency, and (f) cycling stability *via* % capacitive retention of NiO/MgO–SiO_2_ composites (sample 3) as the anode material in the ASC device in 3 M KOH electrolytic solution.

An asymmetric supercapacitor (ASC) based on a two electrode cell set-up was developed in order to assess the practical suitability of the NiO/MgO–SiO_2_ composite as an energy storage device. Activated carbon derived from rice husks was used as the cathode and the NiO/MgO–SiO_2_ composite was used as the anode in the ASC device. Both the cathode and anode have an area of 2 × 2 cm^2^. CV, GCD, and cycling stability were used to study the performance of the ASC device in 3 M KOH electrolytic solution. The CV curves of AC derived from rice husk are shown in the ESI Fig. S2† and they have shown non-Faradic aspects of AC. Fig. 10a and b illustrate the electrochemical performance of the NiO/MgO–SiO_2_ composite at different scan rates and current densities. NiO/MgO–SiO_2_ composites operate within a potential range of −0.1 to 1.6 V without electrolyte polarization. The ASC device has been shown to exhibit pseudocapacitive characteristics both through CV and GCD. As shown in Fig. 10c, the *C*_s_ was calculated using GCD curves at different current densities for the presented ASC device. In the ASC device, it was shown that the *C*_s_ was 344.12 F g^−1^ at 1.5 A g^−1^. Moreover, the scan rate had an impact on the current response and CV curve shape. Fig. 9d shows the energy and power densities calculated from the GCD cycles of the proposed ASC device according to a previous study.^48^ Additionally, the ASC device's cycling stability was investigated *via* repeatable 40 000 GCD cycles, and the device maintained an 88.9% capacitance retention rate at 1.5 A g^−1^ and an 84.6% coulombic efficiency. A slight decrease in cycling stability was observed during the test, which could be attributed to the continuous degradation of the anode and cathode materials. The performance ASC device based on the NiO/MgO–SiO_2_ composite (sample 3) was correlated with the recent ASC devices and it was seen that the proposed ASC exhibited comparable or superior performance to many of the reported ASC devices in terms of synthesis, specific capacitance and cycling stability, as shown in the ESI Table S4.†

## 4. Conclusions

To summarize, grapefruit peel extract was used to synthesize MgO–SiO_2_ composites *in situ*, and different masses were used to fabricate NiO/MgO–SiO_2_ composites through the hydrothermal method. The NiO/MgO–SiO_2_ composites showed the nanoparticle-like orientations and cubic phases of MgO and NiO. Numerous functional groups were observed on the surface of the NiO/MgO–SiO_2_ composites, as well as a significant reduction in the optical band gap of the composites. In alkaline media, these composites were employed as electrocatalytic materials for energy conversion and storage use. The NiO/MgO–SiO_2_ composite (sample 3) exhibited the highest electrochemical performance and the lowest overpotential during the OER activity with significant stability and durability in 1 M KOH. The superior supercapacitor performance of sample 3 was also found to be highly improved, with a specific capacitance of 1248.72 F g^−1^ at 1.5 A g^−1^ in a 3 M KOH solution. An ASC device was constructed using sample 3, which demonstrated an improved specific capacitance of 344.12 F g^−1^, an energy density of 7.31 W h kg^−1^ and a power density of 293.25 W kg^−1^. Over repeated GCD cycles at 1.5 A g^−1^, the enhanced cycling stability of the ASC device based on the NiO/MgO–SiO_2_ composite was also demonstrated with 88.9% capacitance retention. These findings suggest that grapefruit peel extract may serve as a viable biomass waste material for the *in situ* synthesis of MgO–SiO_2_ composites for the development of next-generation energy storage devices.

## Data availability

The authors declare that the data supporting this study's findings are available within the paper.

## Author contributions

Gulzar Ali: conducted material synthesis. Aneela Tahira: performed XRD analysis and drafted the manuscript. Asma Hayat: conducted partial electrochemical tests. Mukhtiar Ali Bozdar: performed FTIR measurements. Muhammad Ali Bhatti: conducted optical band gap analysis. Elmuez Dawi: validated the results. Ayman Nafady: reviewed the obtained results and proofread the manuscript. Matteo Tonezzer: conducted EIS analysis. Ghulam Mustafa Thebo: performed SEM analysis. Muhammad Kashif Samoon: conducted EDS analysis. Zafar Hussain Ibupoto: supervised the work and wrote the original draft of the paper.

## Conflicts of interest

The authors have no conflict of interest in the presented research work.

## Supplementary Material

RA-015-D5RA00671F-s001
