# Supplementary material for: Facile and cost-effective NiO/MgO–SiO2 composites for efficient oxygen evolution reaction and asymmetric supercapacitor systems
Source: RSC Adv. 2025 Mar 6;15(9):7224–35. doi: 10.1039/d5ra00671f (PMC11883754; doi:10.1039/d5ra00671f)
Supplement: RA-015-D5RA00671F-s001 [file RA-015-D5RA00671F-s001.pdf]

Supplementary Information (SI) for RSC Advances.
This journal is © The Royal Society of Chemistry 2025

**Supporting information**

# Facile and cost effective NiO/MgO-$SiO_2$ composites for efficient oxygen evolution reaction and asymmetric supercapacitor systems

Gulzar Ali[1], Aneela Tahira[2], Asma Hayat[1], Mukhtiar Ali Bozdar[8], Muhammad Ali Bhatti[3], Elmuez Dawi[4], Ayman Nafady[5], Matteo Tonezzer[6], Ghulam Mustafa Thebo[7], Muhammad Kashif Samoon[7], Zafar Hussain Ibupoto[*1]

[1]*Institute of Chemistry, University of Sindh, Jamshoro 76080, Pakistan.*
*gulzaralichemist@gmail.com ; asmabaloch141617@gmail.com ; zaffar.ibhupoto@usindh.edu.pk*

[2]*Institute of Chemistry, Shah Abdul Latif University Khairpur Mirs, Sindh, Pakistan. aneela.tahira@salu.edu.pk*

[3]*Centre for Environmental Sciences, University of Sindh Jamshoro, 76080, Sindh, Pakistan. mali.bhatti@usindh.edu.pk*

[4]*College of Humanities and Sciences, Department of Mathematics and Sciences, Ajman University, P.O. Box 346, Ajman, UAE. e.dawi@ajman.ac.ae*

[5]*Chemistry Department, College of Science, King Saud University, Riyadh, 11451, Saudi Arabia. anafady@ksu.edu.sa ; ralshammari@ksu.edu.sa*

[6]*Department of Chemical and Geological Sciences, University of Cagliari, Monserrato, Italy. matteo.tonezzer@cnr.it*

[7]*Centre for Pure and Applied Geology, University of Sindh Jamshoro, Jamshoro, Sindh, 76080, Pakistan. kashif.samoon@usindh.edu.pk*
[8]*Department of Energy and Environment, Sindh Agriculture University Tando Jam, Sindh, Pakistan.*

[*]*Corresponding author(s):*
*Zafar Hussain Ibupoto, PhD, Email: zaffar.ibhupoto@usindh.edu.pk*

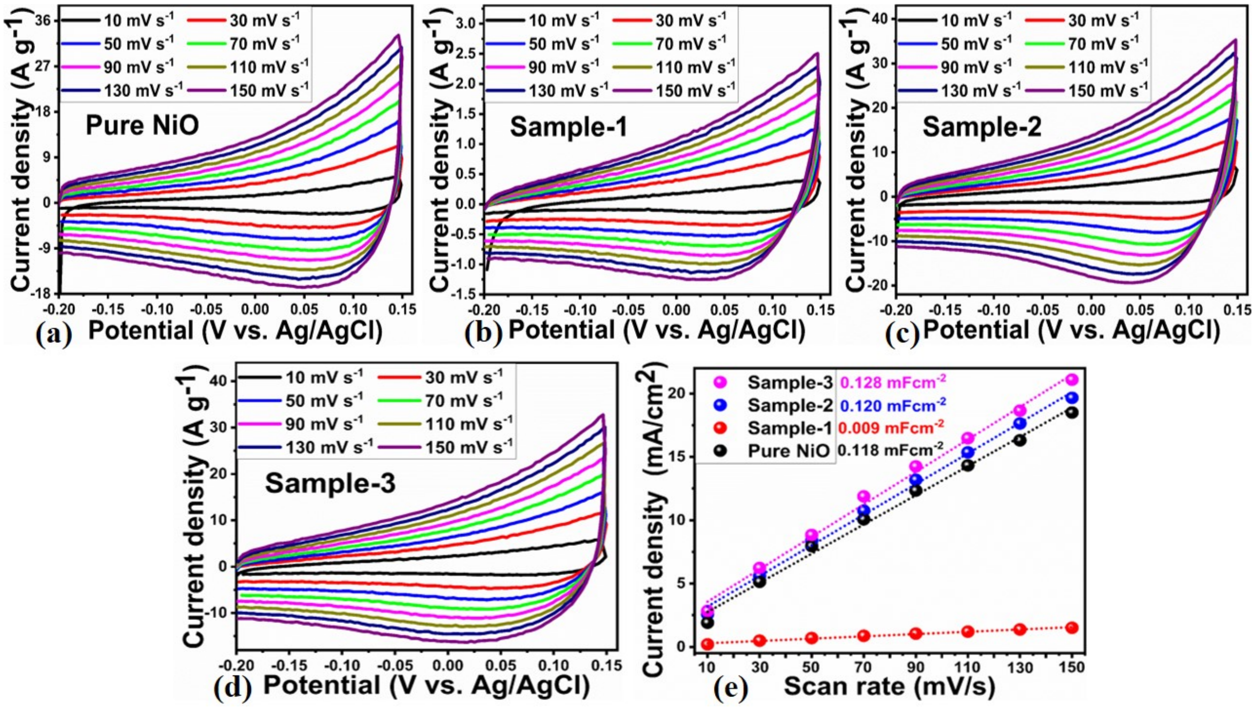


**Figure (S1):** Non Faradic CV curves in 1M KOH aqueous solution using different scan rates (a) pure NiO, (b) MgO-$SiO_2$ composites (sample1), (c,d) NiO/MgO-$SiO_2$ composites (sample 2and sample 3) using different scan rates in electrolytic solution of 3M KOH for the illustration of capacitance performance of each electrode material, (f) Corresponding ECSA analysis.

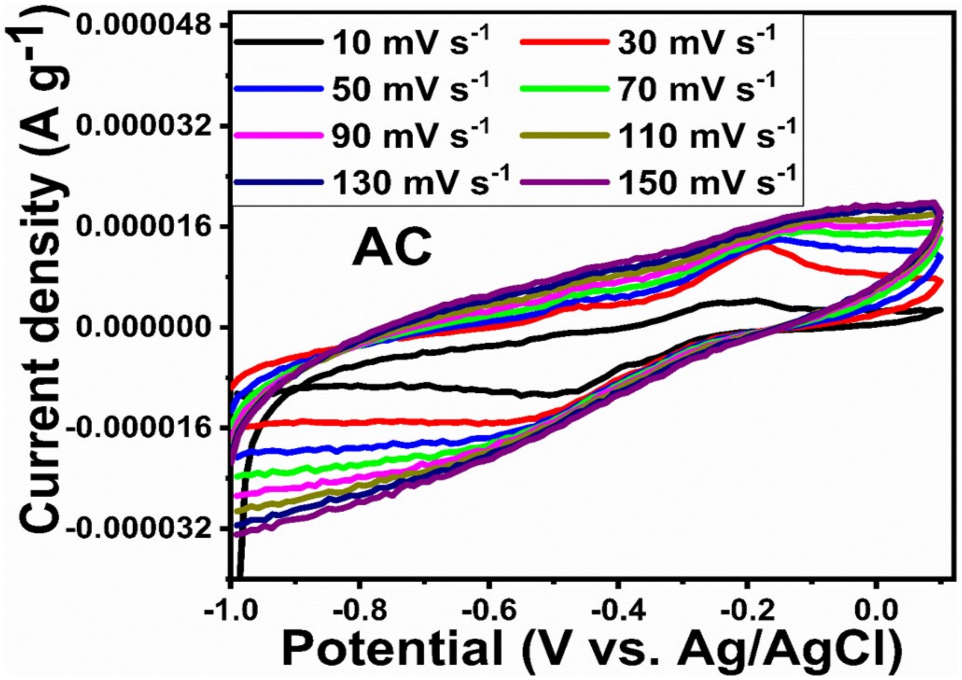

**Figure (S2):** Various CV curves of AC in 3M KOH electrolyte with increasing sweeping rate.

**Table (S1).** OER comparative study of NiO/MgO-$SiO_2$ composite (sample 3).

| Catalyst | η for OER (mV@mA $cm^{-2}$) | Electrolyte | References |
|---|---|---|---|
| $CuCo_2S_4$/$NiCo_2S_4$ | 271 @ 10 | 1.0 M KOH | [1] |
| Mo–Ni–Se@NF | 397@ 100 | | [2] |
| NiCoFe–S/Ti | 230@ 10 | 1.0 M KOH | [3] |
| NiFe/$(Ni,Fe)_3S_2$ | 224@10<br>303@400 | 1.0 M KOH | [4] |
| NiCoP/NF | 280@10 | 1.0 M KOH | [5] |

| | | | |
|---|---|---|---|
| $Co_3O_4$@$CoMoO_4$/NF | 318@20 | 1.0 M KOH | [6] |
| $Co_3O_4$/MgO–$SiO_2$ | 340 @10 | 1.0 M KOH | [7] |
| $Co_3O_4$–MgO | 247@10 | 1.0 M KOH | [8] |
| MgO/Co3O4 (C-2) | 270@10 | 1.0 M KOH | [9] |
| ZnO-Cr-Mg:5% | 345 @10 | 1.0 M KOH | [10] |
| **Sample-3** | **230** | **1.0 M KOH** | **This Work** |


[1] L. Ma, J. Liang, T. Chen, Y. Liu, S. Li and G. Fang, Electrochim Acta. 2019, **326**, 135002.

[2] H. Yang, Y. Huang, W.Y. Teoh, L. Jiang, W. Chen, L. Zhang and J. Yan, Electrochim Acta. 2020, **349,** 136336.

[3] D. Li, Z. Liu, J. Wang, B. Liu, Y. Qin, W. Yang and J. Liu, Electrochim Acta. 2020, **340**, 135957.

[4] C.T. Hsieh, X.F. Chuah, C.L. Huang, H.W. Lin, Y.A. Chen and S.Y. Lu, Small Methods. 2019, **3**, 1900234

[5] H. Liang, A.N. Gandi, A.H. Anjum, X. Wang, U. Schwingenschlögl and H.N. Alshareef, Nano Lett. 2016, **16**, 7718-25.

[6] L. Zhang, T. Mi, M.A. Ziaee, L. Liang and R. Wang, J Mater Chem A. 2018, **6**, 1639-47.

[7]. G. Ali, A. Tahira, A. Hayat, M.A. Bhatti, A.A. Shah, S.N.U.S. Bukhari, E. Dawi, A. Nafady, R.H. Alshammari, M. Tonezzer,M.K. Samoon, Z.H. Ibupoto. 2024. *RSC advances*, 2024, ***14,*** 38009-38021.

[8]. A.J. Laghari, U. Aftab, A. Tahira, A.A. Shah, A. Gradone, M.Y. Solangi, A.H. Samo, M.I. Abro, M. wasim Akhtar, R. Mazzaro, V. Morandi, A. M. Alotaibi, A. Nafady, A. Infantes-Molina, Z. H. Ibupoto, *International Journal of Hydrogen Energy*, **(**2023), *48,* **12672-12682.**

[9]. A.A. Shah, M. Kumar, U. Aftab and M.I. Abro, *Chemical Engineering & Technology*, 2023, ***46***, 459-465.

[10]. B. Naresh, T.V.M. Sreekanth, K. Prasad, K. Yoo and J. Kim, *Surfaces and Interfaces*, 2025, ***56***, 105437.

**Table (S2).** Calculated Supercapacitor parameters of synthesized samples

| Sample | Current Density ($Ag^{-1}$) | Specific Capacitance ($Fg^{-1}$) | Columbic Efficiency (%) | Capacitance Retention (%) |
|---|---|---|---|---|
| Pure NiO | 1.5 | 433.50 | ---------- | ----------- |
| | 2.5 | 161.76 | | |
| | 3.5 | 123.53 | | |
| | 4.5 | 88.62 | | |
| | 5 | 70.33 | | |
| Sample-1 | 1.5 | 414.71 | ---------- | ----------- |
| | 2.5 | 140.66 | | |
| | 3.5 | 97.57 | | |
| | 4.5 | 82.86 | | |
| | 5 | 66.50 | | |
| Sample-2 | 1.5 | 636.83 | 97.6 | 101.5 |
| | 2.5 | 258.95 | | |
| | 3.5 | 196.04 | | |
| | 4.5 | 92.07 | | |
| | 5 | 72.89 | | |
| Sample-3 | 1.5 | 1248.72 | | |
| | 2.5 | 1008.31 | | |
| | 3.5 | 758.18 | | |
| | 4.5 | 253.20 | | |
| | 5 | 148.34 | | |

**Table (S3):** Highlight points of ASC device.

| Sample | Current Density ($Ag^{-1}$) | Specific Capacitance ($Fg^{-1}$) | Energy Density (Wh $kg^{-1}$) | Power Density (W $kg^{-1}$) | Columbic Efficiency (%) | Capacitance Retention (%) |
|---|---|---|---|---|---|---|
| Sample-2 | 1.5 | 344.12 | 7.31 | 293.25 | 84.6 (40000 Cycles) | 88.9 (40000 Cycles) |
| | 2.5 | 275.58 | 5.85 | 488.75 | | |
| | 3.5 | 78.77 | 1.67 | 684.25 | | |
| | 4.5 | 47.19 | 1.00 | 879.75 | | |
| | 5 | 20.46 | 0.43 | 977.50 | | |

**Table (S4).** Comparison of the specific capacitance of $NiO/MgO-SiO_2$ composite (sample 3) with the reported data.

| **Active material** | **Specific capacitance** | **Current density** | **Electrolyte** | **Ref.** |
|---|---|---|---|---|
| $MnO_2/MnCo_2O_4$ | 497 F $g^{-1}$ | 0.5 A $g^{-1}$ | 2 M KOH | 1 |
| $CoMoO_4/NiMoO_4$ | 751 F $g^{-1}$ | 1.0 A $g^{-1}$ | 3 M KOH | 2 |
| $Co_3O_4/NiO/MnO_2$ | 549 F $g^{-1}$ | 0.5 A $g^{-1}$ | 6 M KOH | 3 |
| $CoMn_2O_4$ | 472.6 F $g^{-1}$ | 1.0 A $g^{-1}$ | 2 M KOH | 4 |
| $Co_3O_4$@$MnO_2$ | 1209.4 F $g^{-1}$ | 1.0 A $g^{-1}$ | 2 M KCl | 5 |
| $CoMoO_4$@C@$MnO_2$ | 1824 F $g^{-1}$ | 3.0 A $g^{-1}$ | 3 M KOH | 6 |
| $CuCo_2O_4$@$NiMoO_4$ | 2207 F $g^{-1}$ | 1.25 A $g^{-1}$ | 6 M KOH | 7 |
| $CdMn_2O_4$@$CdMn_2O_4$ | 3885 F $g^{-1}$ | 1.5 A $g^{-1}$ | 2 M KOH | 8 |
| $Co(OH)_2$@$FeCo_2O_4$ | 1173.43 F $g^{-1}$ | 1.0 A $g^{-1}$ | 6 M KOH | 9 |
| Chitosan-modified MgO | 56.32 $Fg^{-1}$ | 0.5 $Ag^{-1}$ | 3 M KOH | 10 |
| NiCo@$Si_1$-C | 518.1 F $g^{-1}$ | 0.5 A $g^{-1}$ | 3 M KOH | 11 |
| RH-$SiO_2$ | 102 F/g | 0.5 A/g | 2 M KOH | 12 |
| $SiO_2$-$NiFe_2O_4$ | 800.8 F $g^{-1}$ | 1 A $g^{-1}$ | 3 M KOH | 13 |
| rGO/n-SiO2 | 166.9 F g | 0.1 A g–1 | 1M $H_2SO_4$ | 14 |
| **Sample-3** | 1248.72 F $g^{-1}$ | 1.5**A $g^{-1}$** | **3 M KOH** | **Present work** |

[1] Y. Zhang, H. Xuan, Y. Xu, B. Guo, H. Li, L. Kang, P. Han, D. Wang and Y. Du, Electrochim. Acta, 2016, **206**, 278-290.

[2] Q. Yang and S.Y. Lin, RSC Adv. 2016, **6**, 10520–10526.

[3] L. Su, Y. Wang, Y. Sha and M. Hao, J. Alloys Compd. 2016, **656**, 585–589.

[4] J. Bhagwan, B.N.V. Krishna and J.S. Yu, Int. J. Energy Res. 2021, **45**, 19413–19422.

[5] K. Wang, Z. Shi, Y. Wang, Z. Ye, H. Xia, G. Liu and G. Qiao, J. Alloys Compd. 2015, **624**, 85–93.

[6] X. Cui, X. Chen, W. Zhang, X. Yan, M. Wang, J. Lian, Z. Zheng and H. Deng, J. Alloys Compd. 2017, **695**, 2109–2116.

[7] J. Lin, H. Liang, H. Jia, S. Chen, Y. Cai, J. Qi, J. Cao and W. Fei, J. Feng, Inorg. Chem. Front. 2017, **4**, 1575–1581.

[8] H.J. Kim, C.W. Kim, S.Y. Kim, A.E. Reddy and C.V.V.M. Gopi, Mater. Lett. 2018, **210,** 143–147.

[9] Z. Wang, P. Hong, S. Peng, T. Zou, Y. Yang, X. Xing, Z. Wang, R. Zhao, Z. Yan and Y. Wang, Electrochim. Acta. 2019, **299**, 312–319.

[10]. S. Sharmila, A. Saranya, S. Rama, M. Lipna, M. Shandhiya, S. Sanjana and A. Murugan, *Ionics*, 2025, 1-20.

[11]. J. Jia, Z. Qin, X. Yang, X. Peng, G. Ren and Z. Lin, *Diamond and Related Materials*, 2023, ***134***, p109786.

[12]. S.R. Priyan, G.S. Kumar, K. Lalithambigai, M. Shkir, A. Khan, R. Rajendran and G. Arumugam, *Journal of Alloys and Compounds*, 2024, ***976***, 173206.

[13]. I.F.F. Christopher, A. Karuppiah, V.G. Thanapalan, A.V. Selestin and T. Suyambu, *Silicon*, 2024, ***16***, 157-176.

[14]. D.D. Ristiana, M. Handayani, M.A. Anggoro, B.W. Widagdo, E. Angelina, H. Sutanto, I. Anshori, E. Febriana, F. Firdiyono, E. Sulistiyono and A.B. Prasetyo, *South African Journal of Chemical Engineering*, 2024, ***48***, 130-137.
